# Modeling Neuroregeneration and Neurorepair in an Aging Context: The Power of a Teleost Model

**DOI:** 10.3389/fcell.2021.619197

**Published:** 2021-03-18

**Authors:** Jolien Van houcke, Valerie Mariën, Caroline Zandecki, Eve Seuntjens, Rajagopal Ayana, Lutgarde Arckens

**Affiliations:** ^1^Laboratory of Neuroplasticity and Neuroproteomics, Department of Biology, KU Leuven, Leuven, Belgium; ^2^Laboratory of Developmental Neurobiology, Department of Biology, KU Leuven, Leuven, Belgium; ^3^Leuven Brain Institute, Leuven, Belgium

**Keywords:** aging, neuroregeneration strategies, neurorepair, zebrafish, killifish, medaka, teleost brain, telencephalon

## Abstract

Aging increases the risk for neurodegenerative disease and brain trauma, both leading to irreversible and multifaceted deficits that impose a clear societal and economic burden onto the growing world population. Despite tremendous research efforts, there are still no treatments available that can fully restore brain function, which would imply neuroregeneration. In the adult mammalian brain, neuroregeneration is naturally limited, even more so in an aging context. In view of the significant influence of aging on (late-onset) neurological disease, it is a critical factor in future research. This review discusses the use of a non-standard gerontology model, the teleost brain, for studying the impact of aging on neurorepair. Teleost fish share a vertebrate physiology with mammals, including mammalian-like aging, but in contrast to mammals have a high capacity for regeneration. Moreover, access to large mutagenesis screens empowers these teleost species to fill the gap between established invertebrate and rodent models. As such, we here highlight opportunities to decode the factor age in relation to neurorepair, and we propose the use of teleost fish, and in particular killifish, to fuel new research in the neuro-gerontology field.

## Introduction

### The Problem: Aging Hampers Neurorepair

According to the United Nations, the number of elderly people aged over 65 years will double worldwide from one to two billion between 2025 and 2075 (World Population Prospects 2019, 2019). This will go hand in hand with an increase in age-related neurological disease states, such as neurodegenerative diseases, stroke, and traumatic brain injury (TBI). In particular, neurodegenerative diseases have become very prevalent in our aging society. The typical progressive loss or dysfunction of specific neurons leads to a multitude of symptoms and deficits, including memory loss, motor impairment, and behavioral changes (Dugger and Dickson, 2017). These diseases heavily reduce the life quality of the patient and represent a high social and economic burden (El-Hayek et al., 2019). Developing treatments that can prevent, cure, or slow down their progression is of utmost importance yet challenging. Despite major efforts, there are still no effective treatments available to restore injury- or pathology-induced neuronal death and dysfunction (Han et al., 2016; Shohayeb et al., 2018). In part, pathology should be alleviated by the replacement of dead or dysfunctional neurons by new ones, that is neuroregeneration, or by the protection of the damaged and newly formed neurons to withstand degeneration, that is neuroprotection. The capacity to fully and successfully replace neurons is, however, limited in the adult mammalian brain. The non-permissive environment fails to give trophic support, causing misplacement, malformation, or death of the newly formed neurons (Arvidsson et al., 2002; Villasana et al., 2015; Ibrahim et al., 2016). Moreover, the already limited neuroregenerative capacity declines even further with age, which is the number one risk factor for neurodegenerative disease (Apple et al., 2017). Aging alters cell-intrinsic and cell-extrinsic factors (the microenvironment) of stem cells, leading to reduced neurogenesis, differentiation, and integration of newborn neurons (Duan et al., 2003; Decarolis et al., 2015; Apple et al., 2017; Wang et al., 2017).

### Teleost Fish: The Best of Two Worlds

Considering the pleiotropic nature and the late onset of neurodegenerative diseases, current research focusing on finding neuroreparative therapies is in need of reliable aging models (Johnson, 2015). Here, teleost fish seem to come in handy. Several teleost species, such as medaka (*Oryzias latipes*), guppy (*Poecilia reticulata*), zebrafish (*Danio rerio*), and turquoise killifish (*Nothobranchius furzeri*), have gained much attention in gerontology research (Ding et al., 2010; Gopalakrishnan et al., 2013; Van houcke et al., 2015; Kim et al., 2016; Platzer and Englert, 2016; Ackerman and Gerhard, 2018). While sharing the feasibility of large-scale mutagenesis analysis with most invertebrate models, teleosts are more directly relevant to human biology and disease modeling. Moreover, knowledge about the robust regenerative properties of teleost fish may help in addressing the limited neurogenic capacities of mammalian models. It seems that at least some teleost species may hold the two key features needed to boost the quest for effective therapies to combat the impact of aging on the brain: a robust regenerative capacity and an aging process that resembles that of mammals. Understanding how teleost fish retain or lose their regenerative ability upon aging will be valuable in designing novel therapies that can boost successful neurorepair, even in aged patients, including those suffering from neurodegenerative disease.

**FIGURE 1:**
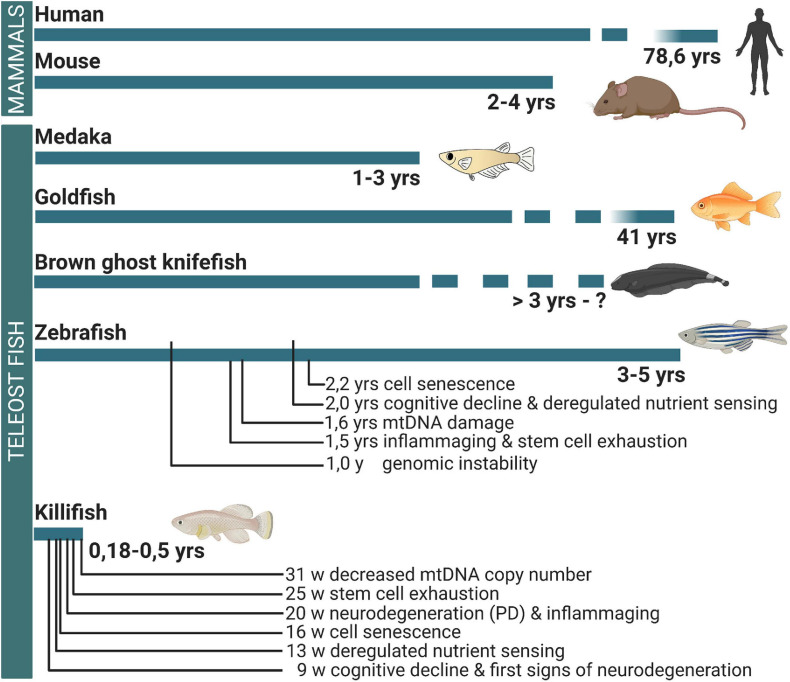
Differences in the lifespan between mammals and teleost fish and the occurrence of brain aging hallmarks. While medaka, goldfish, brown ghost knifefish, and zebrafish have a lifespan longer or comparable to mouse, the killifish has a mean lifespan that is eight times shorter. In the killifish brain, cellular and molecular aging hallmarks are already observed from 9 weeks of age. For comparison, the occurrence of important hallmarks of brain aging is linked to the timelines, based on observations in zebrafish and killifish. Yrs, years; W, weeks; PD, Parkinson’s disease-like phenotype; MtDNA, mitochondrial DNA.

In this review, we put a new perspective on the use of teleost fish to study age-related neuropathology. We discuss data that demonstrate the existence of mammalian aging hallmarks in the teleost brain, assuring that teleosts can be used as gerontology models for human disease. Next, we elaborate on current knowledge about how teleost fish retain or lose their neuroregenerative abilities upon aging. Moreover, we emphasize the African turquoise killifish as the most convenient model among these different species. Indeed, most teleosts are still relatively long-lived (>3 years, Figure 1), just like mice, making gerontology research costly and slow (Gerhard et al., 2002; Miller et al., 2002; Gopalakrishnan et al., 2013). The African turquoise killifish can help in circumventing this. When bred in captivity, this small teleost has a maximum lifespan of 12–60 weeks depending on the strain or rearing conditions and shows typical aging hallmarks (Valdesalicil and Cellerino, 2003; Wendler et al., 2015; Kim et al., 2016; Platzer and Englert, 2016). We will therefore illustrate whenever possible based on current knowledge that especially neuroresearch in killifish holds great potential for future developments of new therapies for age-related brain disease.

## Evidence for Molecular and Cellular Hallmarks of Aging in the Teleost Brain

Aging can broadly be described as the gradual deterioration of tissues and organs that eventually leads to a decline in biological functioning or fitness. Reversing this deterioration has been a topic of interest for many years. Aging is, however, pleiotropic in nature and cannot be reversed by a simple on/off switch mechanism. Using different animal gerontology models can therefore help to disentangle the many facets of aging and its characteristics. López-Otín et al. (2013) were the first to elegantly portray nine interconnected cellular and molecular hallmarks of aging, focusing on mammals, that together determine the aging process. Genomic instability, epigenetic alteration, telomeric attrition, and loss of proteostasis are primary hallmarks of aging since they act as the initial triggers of the aging process. Eventually, they cause the appearance of antagonistic hallmarks, which are beneficial or deleterious depending on their intensity. This is true for deregulated nutrient sensing, mitochondrial dysfunction, and cellular senescence (López-Otín et al., 2013). The primary and antagonistic hallmarks eventually result in changes in the phenotype, called the integrative hallmarks: altered intercellular communication and stem cell exhaustion (López-Otín et al., 2013).

Although it has been clear for some time that most of the nine hallmarks also apply body-wide in teleost fish (Van houcke et al., 2015; Platzer and Englert, 2016), a lot of new and exciting research has been performed recently. We will therefore discuss each of the nine aging hallmarks by highlighting the existing literature as an illustration of their occurrence specifically in the brain of aged teleosts. We will focus on zebrafish and killifish and, where appropriate, also discuss the far more sparse findings in medaka, goldfish, brown ghost knifefish, guppy, kokanee salmon, and rainbow trout. As such, we set the stage for the aged teleost brain to be used as a gerontology model for age-related neuropathology and neurorepair.

### Genomic Instability

Throughout life, DNA endures a lot of stress that can be either exogenous in nature, as with physical, chemical, and biological agents, or endogenous, as with reactive oxygen species production and DNA replication flaws. These stress factors can eventually lead to genetic lesions that accumulate over time. Although the cell contains repair mechanisms, these are often erroneous, creating genomic instability and disease (Best, 2009; Vijg and Suh, 2013). The zebrafish brain shows nuclear and mitochondrial DNA damage (fragmentation and lesions) already at a young age. From the age of 12 months onward, this damage steeply increases (Shimoda et al., 2014; Zhu and Coffman, 2017). In the adult mammalian and teleost brain, segregation defects during division can result in aneuploidy, a non-diploid chromosome content in cells (Rajendran et al., 2007, 2008; Westra et al., 2008; Zupanc et al., 2009). Loss or gain of chromosomes after division is suggested to be linked to neurodegenerative disorders (Faggioli et al., 2011). On the contrary, aneuploidy is regularly observed during mammalian development and into adulthood (Westra et al., 2008). Also, in the adult zebrafish and brown ghost knifefish brain, newborn neurons with segregation defects are shown to mature and are long-lived (Rajendran et al., 2007, 2008; Zupanc et al., 2009). The loss or gain of chromosomes in newborn cells might thus be a normal biological process for the genomic diversification of newborn neurons and glia. In the mouse cerebral cortex, aneuploidy increases upon aging (Faggioli et al., 2012). For teleost fish, this remains to be found.

### Epigenetic Alterations

The epigenome can alter the availability and stability of genetic information written in DNA. Epigenetic changes include DNA methylation, histone modifications, chromatin remodeling, histone type, etc. With age, the epigenome configuration can be lost or altered, eliciting changes in gene transcription. It can thus explain how two individuals with the same genome, i.e., identical twins, develop and age in a different way. Surely, the epigenome can be influenced by exogenous and endogenous factors (Pal and Tyler, 2016). In the killifish brain, Hdac1 and Hdac3, genes with a function in chromatin remodeling, more specifically histone modification, are significantly downregulated upon aging (Zupkovitz et al., 2018). In addition, the zebrafish brain shows age-related hypomethylation (Shimoda et al., 2014). These reports indicate that (epi)genomic instability increases with age in the teleost brain, just like in mammals (López-Otín et al., 2013).

### Telomeric Attrition

Telomeres are tandem repetitive DNA sequences at the end of chromosomes. They protect the chromosome ends of being recognized as double-stranded breaks by the DNA damage repair response (DDR), but fail to do so when shortened in the context of cell division. As a consequence, the cell goes into replicative senescence *via* DDR pathways (Zhu et al., 2019). Attrition of telomeres can, however, be counteracted by telomerase, a ribonucleoprotein complex containing a telomerase reverse transcriptase (TERT) and telomerase RNA, that can lengthen the telomeres again at each cell division (Zhu et al., 2019). In most mammalian adult somatic tissues, telomerase expression is, however, insufficient to rescue telomeres from attrition (Collins and Mitchell, 2002; Zhu et al., 2019). In several zebrafish tissues, including the skin, eye, and gill, TERT expression decreases from 12 months onward. In the brain, only a decreasing trend could be observed (Anchelin et al., 2011), yet aged zebrafish brains (26–32.5 months old) also show a trend in shortening of the telomeres (Arslan-Ergul et al., 2016). Hence, decreased TERT expression could possibly lead to telomere shortening in the zebrafish brain with age. Nonetheless, many studies still show contradictory results (Simide et al., 2016). In killifish, age-related telomere shortening has only been studied in the muscle and gill, where the long-lived MZM-04/10, but not the short-lived GRZ, killifish strain demonstrates telomeric attrition upon aging, despite increasing TERT expression with age (Hartmann et al., 2009). In the liver of *Nothobranchius guentheri*, telomerase activity remained constant between different age classes (Liu et al., 2012). More research is thus needed to define if the teleost brain is susceptible to telomeric attrition and its implications.

### Loss of Proteostasis

As a consequence of the loss of genomic and epigenetic stability with age, protein homeostasis, called proteostasis, gets altered upon aging. Proteostasis relies on the protein control mechanisms of the cell, including the stabilization of correctly folded proteins and the degradation of aberrant proteins. Together, they act against the occurrence of protein aggregates that are typically associated with (neuro)degeneration and age-related cognitive decline (Hipp et al., 2019). Indeed, cognitive ability, such as associative and spatial learning, declines in aged zebra- and killifish (Valenzano et al., 2006a, b; Ruhl et al., 2015). In aged zebrafish (2 years old), these deficits appear at the time when oxidized proteins and lipofuscin accumulate in the dorsal telencephalon (Ruhl et al., 2015). Lipofuscin is an aggregated autofluorescent lipopigment, containing oxidized lipids and proteins, and increases in cells with age and is thus often used as a marker for aged cells (Seehafer and Pearce, 2006). Lipofuscin has been shown to inhibit the proteosomal system (Höhn et al., 2011) and, hence, to further exacerbate loss of proteostasis. Also in the aged killifish brain (39 weeks old, long-lived MZM 04/10 strain), imbalance in stoichiometry of major protein complexes was found together with aggregation of ribosomes and a reduced proteasome activity (Sacramento et al., 2020). Besides aggregated ribosomes, increased α-synuclein deposition has been demonstrated in the aged killifish brain (Matsui et al., 2019). Linked to this, dopaminergic neurons in the posterior tuberculum and noradrenergic neurons of the locus coeruleus appear to be degenerated in the aged killifish brain (aged 5 months, MZCS 2010/24 strain), which is not the case for zebrafish or medaka (aged 1 year) (Matsui et al., 2019). Killifish thus seem to spontaneously develop a cellular Parkinson’s disease phenotype similar to that observed in humans (Matsui et al., 2019). The question arises if a 1-year-old zebrafish or medaka is comparable to a 5-month-old killifish, since their lifespans differ substantially (Figure 1). The neurodegenerative phenotype is, however, eminent in killifish and can be studied within a practical short timeframe (Figure 1). Indeed, Fluoro-Jade B staining, a marker for neurodegeneration, is increased in the aged killifish optic tectum and cerebellum when compared with young fish (Valenzano et al., 2006b; Liu et al., 2012). Besides killifish, age-related neurodegenerative phenomena have also been reported in the optic tectum of aged guppies (>2 years old) and in aged brains of the kokanee salmon (approximately 3–4 years old) (*Oncorhynchus nerka kennerlyi*) (Woodhead and Pond, 1984; Maldonado et al., 2000, 2002a, b). These results convincingly demonstrate the power of teleost fish and, in particular, killifish as a model for age-related neuropathology due to loss of proteostasis.

### Deregulated Nutrient Sensing

The insulin/IGF-1 signaling pathway is the most conserved aging controlling pathway in evolution. Two of its important targets, the FOXO family and mTOR pathway, are involved in aging and longevity (López-Otín et al., 2013; Farr and Almeida, 2018). Dietary restriction without malnutrition or reduced functioning of the insulin/IGF-1 pathway can extend the lifespan in organisms (Fontana, 2009; Arslan-Ergul et al., 2013). Out-of-proportion downregulation of the pathway, however, exacerbates the aging process (López-Otín et al., 2013). The zebrafish aged brain (31–36 months old) shows downregulation of *igf1*, *igf2bp3*, and *igfbp2a*, which are important players in IGF-1 signaling (Arslan-Ergul and Adams, 2014). Furthermore, within the cell surface proteome of radial glia, the IGF-1 receptor is downregulated in the aged zebrafish telencephalon (2 years old) (Obermann et al., 2019). Sirtuins (*sirt*) are other important nutrient sensors that detect low energy states and are implicated in lifespan regulation (López-Otín et al., 2013). The aged killifish brain (13 weeks old) shows upregulation of *sirt1* and *sirt7* and downregulation of *sirt3* (Kabiljo et al., 2019). Moreover, when aged killifish (9 weeks old, GRZ strain) are treated with the *sirt2* activator resveratrol, these fish have a longer lifespan, improved active avoidance learning, and decreased neurodegeneration in the optic tectum, as visualized by reduced Fluoro-Jade B staining (Valenzano et al., 2006b, Yu and Li, 2012). As such, there is increasing proof that altered nutrient sensing with age is occurring in the teleost brain. Researchers have taken advantage of this aging hallmark to test the effect of dietary restriction (DR) on longevity and the senescence brain phenotype. Short-term DR decreases body weight but not body length (similar growth) or cortisol levels (similar stress levels) in both young (8–8.5 months old) and aged (26–32.5 months old) zebrafish. When testing if DR zebrafish had a similar senescence phenotype than *ad libitum*-fed control zebrafish, the results were contradictory. Instead of the expected elongation or preservation, telomeres in the zebrafish brain appear shortened upon DR treatment. In addition, there is no improvement in cell proliferation and no decrease in the amount of senescent cells present in the telencephalon of young and aged DR zebrafish (Arslan-Ergul et al., 2016). The killifish brain, on the contrary, does demonstrate improvement of age-related cognitive decline after DR. Old DR killifish (9 and 11 weeks old, GRZ strain) have a longer lifespan, improved active avoidance learning, reduced lipofuscin accumulation in the liver, and less neurodegeneration in the brain as tested with Fluoro-Jade B staining when compared with *ad libitum*-fed control killifish (Terzibasi et al., 2009). DR also elicits upregulation of GFAP in the killifish brain (Terzibasi et al., 2009), a marker of reactive gliosis and associated with radial glia proliferation after injury (März et al., 2011), but also with aging (Terzibasi Tozzini et al., 2012). It would therefore be relevant to test if such DR killifish would display more dividing stem/progenitor cells than killifish on standard diet. However, treatment of zebrafish with rapamycin, an mTOR inhibitor that mimics DR, lowered GFAP expression in the aged brain, but not in the young brain (Saxton and Sabatini, 2017; Celebi-Birand et al., 2020). Likewise, only in young fish, rapamycin administration lowered PCNA levels in the brain (Celebi-Birand et al., 2020). It seems that the type of treatment, the length of treatment, and the age of the animals influence the outcome of DR.

### Mitochondrial Dysfunction

Aberrant mitochondrial functioning increases with age and is characterized by loss of efficiency in the electron transport chain, reduced generation of high-energy molecules (ATP), and increased reactive oxygen species (ROS) production and mutations in mtDNA (López-Otín et al., 2013). Although an out-of-proportion amount of ROS is generally considered as detrimental for cell integrity, low levels can elicit proliferative and survival signals (Sena and Chandel, 2012). In the teleost central nervous system (CNS), there are several signs of age-related mitochondrial dysfunction. The aged zebrafish retina (19 months old), for instance, shows decreased mitochondrial integrity and DNA copy number as well as altered expression of key players of mitochondrial biogenesis (Wang et al., 2019). Likewise, the aged brain of zebrafish (20 months old) and killifish (31 weeks old, long-lived MZM-0403 strain) display a higher amount of mtDNA lesions and a decrease in mtDNA copy number, respectively (Hartmann et al., 2011; Zhu and Coffman, 2017). Also, the membrane phospholipid composition of the mitochondria was found altered with age in the brain and heart of the rainbow trout (*Oncorhynchus mykiss*) (Almaida-Pagán et al., 2012). Although more research is needed, these studies already hint toward mitochondrial dysfunction in the aged teleost brain.

### Cellular Senescence

Among others, genomic lesions, telomeric attrition, and oxidative stress corrupt the cell as it ages. To avoid malignant transformation, the cell goes into an arrested or dormant state, called senescence (Chinta et al., 2015). While senescence thus protects against cancer, it is often deleterious in older organisms. Senescent cells have a senescence-associated secretory phenotype (SASP), which includes the secretion of inflammatory cytokines, chemokines, growth factors, and proteases. In a way, this is beneficial since the SASP serves as a signal for the removal of senescent cells by the immune system. On the contrary, when not removed, SASP factors create a sustained inflammatory environment that is suspected to abate tissue integrity (Chinta et al., 2015). In addition, to retain homeostasis, removed senescent cells will also need to be replaced by new ones by inducing progenitor proliferation. Yet, also this process turns less efficient with age, when stem cell pools get depleted and exhausted (López-Otín et al., 2013). Cell senescence can be visualized *via* biomarkers, including the expression level of cell cycle inhibitors, e.g., p16 and p21, and SASP factors, e.g., IL6, IL8, CCL2, and MMP-1, among others (Hernandez-Segura et al., 2018). To our knowledge, increased expression of such factors has not yet been reported in the aged teleost brain. Nevertheless, a significant amount of senescence-associated β-galactosidase (SA β-gal) activity is observed in the aged telencephalon of both zebrafish (Arslan-Ergul et al., 2016) and killifish (16 weeks old, short-lived GRZ strain, Van houcke, J. unpublished results). SA β-gal is the most extensively used biomarker for replicative or induced senescence because of its easy use. The β-gal protein increases with age in lysosomes. Yet, SA β-gal activity is not necessary for senescence, but rather an outcome (Lee et al., 2006). The increase of SA β-gal activity with age in the teleost brain reveals the accumulation of senescent cells and confirms the existence of this hallmark in the aged teleost brain.

### Altered Intercellular Communication

Aside from cell-autonomous changes, intercellular communication at the endocrine, neuroendocrine, or neuronal level changes with age. Indeed, the radial glia cell surface proteome of aged zebrafish brains shows downregulation of several components linked to N-cadherin signaling (DAGLA, CDH2, CDC42, CTNNA1, KIF5B), suggestive of altered intercellular interactions (Obermann et al., 2019). Also, cytokine–cytokine receptor interactions and Jak–Stat signaling are modified upon brain aging in killifish (long-lived MZM-0410 strain) (Baumgart et al., 2016). One of the most profound changes of the aged brain is chronic inflammation, also called inflammaging. Such sustained inflammation decreases tissue integrity and is highly linked to neurodegenerative disease. Multiple theories considering the mechanisms of inflammaging are summarized in Xia et al. (2016). Aged zebrafish (18 months old) show a higher number of microglia, both ramified and round, in the telencephalon compared with young fish (Bhattarai et al., 2017). The aged killifish telencephalon (5 months old) also has more apoEb-positive microglia compared with young (1 month old) individuals (Matsui et al., 2019). Elevated transcripts of inflammation were also observed in the aged killifish brain (Petzold et al., 2013). Inflammaging seems thus present in the aged teleost brain, suggesting that teleost fish indeed also display aging-altered intercellular communication.

### Stem Cell Exhaustion

The multiple levels of damage caused by the hallmarks described above eventually also influence the intrinsic capacity of stem cells to proliferate and generate new neurons, glia, or neural stem cells (Nicaise et al., 2020). As in the adult mammalian brain, neurogenesis in the teleost brain declines with age (Terzibasi Tozzini et al., 2012; Edelmann et al., 2013; Nicaise et al., 2020). In the zebrafish olfactory bulb, the number of newborn neurons decreases steeply between 3 and 6 months of age, holds constant levels until 10 months of age, and then diminishes further. Also, in the zebrafish telencephalon, the number of newborn neurons and oligodendrocytes shows an age-related decline (Edelmann et al., 2013). Stem cell exhaustion is likewise observed in the killifish telencephalon where immunoreactivity for doublecortin, a marker for neuroblasts and newborn neurons, markedly decreases upon aging (Terzibasi Tozzini et al., 2012). Brown ghost knifefishes represent an exemption to this as they only show very mild decreases in stem cell proliferation and neuron/glia production with advancing age (Traniello et al., 2014). Like zebrafish and killifish, the brown ghost knifefish keeps on growing after reaching maturity, but it does so without reaching a plateau at old age or losing gonadal mass (Ilieş et al., 2014). This teleost species thus models negligible senescence instead of showing gradual senescence like in zebrafish (Kishi, 2004).

Taken together, these reports clearly provide mounting evidence that the nine mammalian hallmarks of aging also characterize the aging teleost brain, which therefore can be used as a model for age-related neuropathology. In particular, killifish grasp our attention because these fish have a more pronounced mammalian-like phenotype in a very short lifespan when compared with zebrafish, e.g., spontaneous age-related neurodegeneration and improvement of the aging phenotype by dietary restriction. Yet, some teleost fish, e.g., the brown ghost knifefish, show a remarkable resilience to aging and may present a valuable model to understand how this resilience is molecularly and cellularly different from other teleost fish or mammals.

Different teleost species thus seem to display a spectrum of vulnerability to aging. Side-by-side comparison of teleost models of negligible aging and rapid aging might help in revealing key concepts about the biology of aging and the evolution of senescence. In addition, comparison of the genetic diversity of closely related long- and short-lived species or strains might help in disentangling chronological aging from biological aging (Petzold et al., 2013; Baumgart et al., 2015). Killifish are particularly useful in such comparisons since fish derived from arid regions in Africa have shorter lifespans than fish derived from humid habitats (Genade et al., 2005; Terzibasi et al., 2008; Terzibasi Tozzini et al., 2013). In captivity, the respective lifespan of these populations largely remains. Similar approaches already revealed several genes involved in lifespan regulation (Reichwald et al., 2015; Valenzano et al., 2015; Sahm et al., 2017). The short-lived laboratory killifish strain is also appealing for longitudinal studies and studies that want to track life-history traits over multiple generations. Its rapid growth and rapid aging leading to signs of age-related neurodegeneration in the brain already by 9 weeks of age seem to position the short-lived GRZ strain (*N. furzeri*) as an outlier for neurodegeneration with respect to other teleost fish (Valenzano et al., 2006b). We predict that the short-lived killifish will be of excellent value to bridge the gap between extremely short-lived invertebrate models (i.e., *Caenorhabditis elegans*) and long-lived mammalian model organisms currently used in the quest for better treatments for age-related brain diseases.

## Impact of Aging on the Neuroregenerative Capacity in the Adult Teleost

### The Regeneration Capacity of the Teleost Fish Brain

The adult vertebrate CNS harbors neural stem cells (NSCs) in active neurogenic niches that generate new neurons throughout life. This ability gives the CNS a certain plasticity to reshape some neural circuits and likewise respond to pathology or injury by replenishing the lost cell types, called regeneration. The capacity to fully and successfully regenerate, however, differs across species, although the molecular and cellular mechanisms of the regeneration process are quite similar (Alunni and Bally-Cuif, 2016).

**FIGURE 2:**
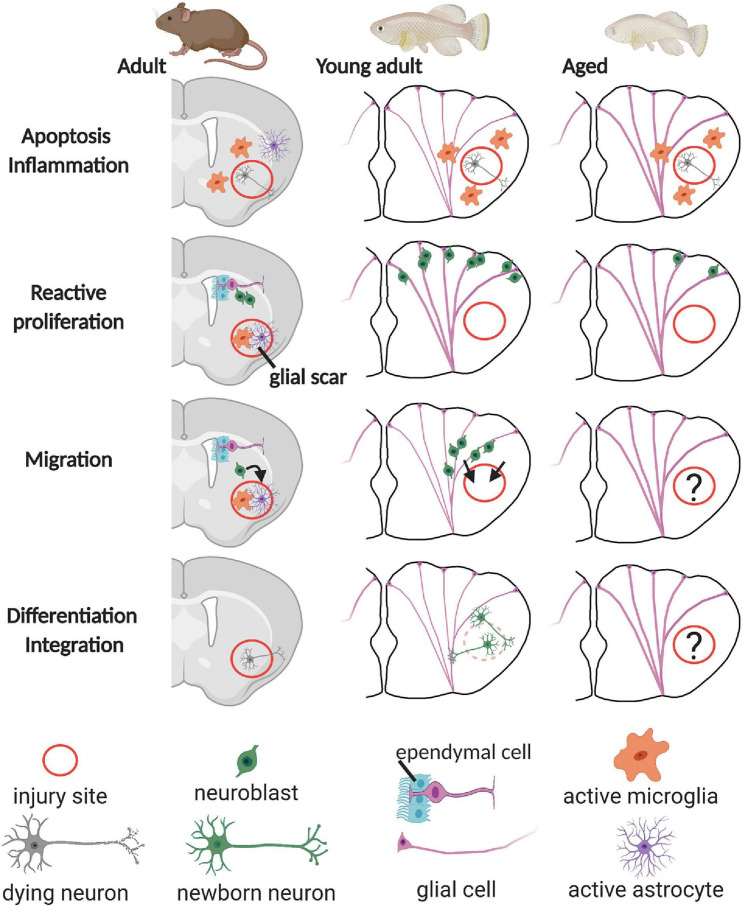
Overview of the regeneration process in the rodent, young teleost, and aged teleost pallium. After injury, neural stem cells with glial characteristics (glial cell), located at the ventricular surface, start proliferating and produce neuroblasts that can replenish the lost neurons. In the rodent pallium, the inflammatory reaction and the formation of a glial scar inhibit proper integration and survival of the newborn neurons into the existing circuitry. In the young adult teleost pallium on the contrary, newborn neurons mature, integrate, and are long-lived. Whether this is also the case for the aged teleost pallium remains elusive (question marks). Note that thicker glial fibers represent that radial glia are activated in the young teleost telencephalon. The ongoing inflammaging process in the aged teleost brain is visualized by thick glial fibers–activated radial glia in all panels.

In the mammalian brain, severe injuries, e.g., stroke, TBI, epilepsy, and Huntington’s disease, lead to the production of neuroblasts that migrate from the neurogenic regions to ectopic sites, the injury site. Upon arrival, newborn neurons are found to form synaptic contacts for proper integration in the neural circuit. However, the vast majority of the newborn neurons die after some time (Figure 2). It remains unclear for now if this is caused by a non-permissive inflammatory environment, an intrinsic failure of newborn neurons to integrate, or a combination of both (Kernie and Parent, 2010; Grade and Götz, 2017). After brain injury or pathology, the immune system elicits a neuroinflammatory response, which has been shown to have contradictory effects. Generally, the inflammation response, mediated by pro-inflammatory microglia and macrophages, is meant to signal NSCs to produce neuroblasts and to provide trophic support. In addition, local inflammation promotes the formation of a glial scar, consisting of reactive astrocytes, microglia/macrophages, and extracellular matrix proteins that seal the injury site (Figure 2). The scar prevents further damage to the tissue, restores homeostasis, and coordinates immunity. In the later stages, however, if the scar is not resolved, it blocks the growth of newborn neurons (axonal regeneration) and subsequently inhibits overall repair (Martino et al., 2011).

In contrast to mammals, teleost newborn neurons can integrate in the existing circuitry and are long-lived, and the formation of a glial scar is rarely observed in the injured CNS (Figure 2; Kroehne et al., 2011; Baumgart et al., 2012; Skaggs et al., 2014). Furthermore, teleost fish have numerous neurogenic zones dispersed over the brain, as described in the guppy (*P. reticulata*) (Kranz and Richter, 1970a, b; Richter and Kranz, 1970a, b), the brown ghost knifefish (*Apteronotus leptorhynchus*) (Zupanc and Horschke, 1995), the gilt-head sea bream (*Sparus aurata*) (Zikopoulos et al., 2000), the three-spined stickleback (*Gasterosteus aculeatus*) (Ekström et al., 2001), the zebrafish (*D. rerio*) (Grandel et al., 2006), the annual killifish (*Austrolebias* sp.) (Fernández et al., 2011), the African turquoise killifish (*N. furzeri*) (Terzibasi Tozzini et al., 2012), and medaka (*O. latipes*) (Kuroyanagi et al., 2010). Also, the daily production rate of new cells is higher compared with mice or rats (Zupanc and Sirbulescu, 2011). All these aspects thus likely lie at the basis of the high neuroregenerative capacity of the teleost CNS.

### Aging and the Regeneration Capacity of Teleost Fish: Retained or Lost?

Teleost fish thus have a high neurogenic capacity but can also display mammalian-like brain aging characteristics. Consequently, the question arises if their ability for neurorepair is retained or either lost with age, as found in mammals. Both scenarios open up intriguing avenues for future research. If they retain the regenerative ability upon aging, it will be of major importance to find out how they do so and whether this asset can be translated to mammals, in relation to the 70% gene conservation between teleost fish and mammals (Howe et al., 2013). If the regenerative capacity is reduced or lost, teleost fish can represent a robust model in which large-scale mutagenesis and drug screenings can be performed, acting as a bridge between short-lived invertebrate and long-lived mammalian aging research. In this next section, we will point out what is currently known about how aging changes neurorepair in teleosts, which, for now, is mostly studied in zebrafish.

In particular, we will discuss regeneration in the young adult and aged teleost telencephalon, as this brain area is the most relevant for comparative study to mammalian brain regeneration. For regeneration studies in other parts of the teleost CNS, we kindly refer to other publications on the diencephalon (Vijayanathan et al., 2017; Caldwell et al., 2019), cerebellum (Zupanc et al., 1998, 2003, 2006; Zupanc and Ott, 1999; Clint and Zupanc, 2001, 2002; Ilieş et al., 2012), retina (Hitchcock and Raymond, 2004), and spinal cord (Ghosh and Hui, 2018). Indeed, the teleost telencephalon holds the subpallial and pallial neurogenic niches of the telencephalon, which are thought to be homologous to the mammalian subventricular zone and the subgranular zone – the two main neurogenic niches in adult mammals (Adolf et al., 2006; Grandel et al., 2006; Broglio et al., 2010; Durán et al., 2010; Ganz et al., 2010, 2014). The NSCs in these neurogenic niches run along the ventricle, also called the ventricular zone (VZ), and cycling NSCs give rise to neurons that typically migrate only one or two cell sizes away from the VZ, ending up in what is called the periventricular zone (PVZ) (Adolf et al., 2006; Grandel et al., 2006).

### Neuroregeneration in the Young Adult and Aged Telencephalon

In young adult zebrafish, the injured telencephalic parenchyma has a spongy appearance, a measurable parameter for the injury perimeter, and an indicator of local intra- and extracellular edema. This spongy appearance diminishes already within the first 7 days (Kroehne et al., 2011; März et al., 2011). Around the injury site, damaged neurons and other cells undergo apoptosis as soon as 4 h post injury (hpi) until 3 days post injury (dpi) (Kroehne et al., 2011). Surprisingly, in an Aβ42 toxicity injury model (amyloidosis), the apoptotic response is similar between young (6 months old) and aged zebrafish (18 months old) (Bhattarai et al., 2017), while aging is expected to increase cellular vulnerability and increase the likelihood to undergo cell death after injury (Itoh et al., 2013; Sun et al., 2013). Aged Aβ42-treated zebrafish do, however, show a larger loss of synapses in the VZ of the telencephalon in comparison with young Aβ42-treated fish. In the parenchyma, the magnitude of lost synapses is, however, similar between young and aged Aβ42-treated zebrafish (Bhattarai et al., 2017). These results thus only partly demonstrate that aging can exacerbate the outcome of induced neuropathology yet highlight the need to take an aging environment into account in neurodegenerative disease model systems. In addition, it would be most interesting to include zebrafish beyond the age of 18 months in future research to elucidate if more clear effects on apoptosis and synaptic degeneration can be discovered at a higher age.

The young and aged Aβ42-treated zebrafish also show an inflammatory response, which is larger in young zebrafish, because aged zebrafish already have more activated microglia (round L-plastin-positive cells) in naive conditions, a strong indicator of inflammaging. Bhattarai et al. (2017) hypothesize that microglia activation protects synapse integrity in young fish. Acute inflammation indeed has many positive effects on brain injury in teleost fish. Macrophages and microglia remove dead cells and cell debris to clear out the injury wound. Furthermore, acute inflammation is also a signal for radial glia, the common NSCs of the zebrafish brain, to activate (Kyritsis et al., 2012; Bhattarai et al., 2016). When the anti-inflammatory drug dexamethasone was given to injured young zebrafish, reactive proliferation was significantly diminished, and as a consequence, fewer neurons were born (Kyritsis et al., 2012). Inflammation is thus a positive effector of regeneration in the young teleost telencephalon while mostly found detrimental for regeneration in mammals. In aged teleost fish as well, it seems that the inflammatory reaction is insufficient to prevent synaptic degeneration. Elucidating how aged teleost microglia differ from young microglia could therefore deliver the knowledge needed for effective tweaking of the inflammation response in the aged mammalian brain to install successful regeneration.

Acute inflammation thus activates NSCs in the telencephalon after injury. In zebrafish, the NSC population that reacts most to the injury is the radial glia, approximately 86.5 ± 3.1% (*n* = 4) of all dividing cells at 3 dpi (Kroehne et al., 2011). The other dividing NSC population has no glial markers and is suggested to be committed progenitors at the ventricle (März et al., 2011). In the aged zebrafish telencephalon (28 months old), proliferation of radial glia is increased after injury, but less profound compared with the young (8 months old) telencephalon (Figure 2). Approximately 18 versus 40% of all radial glia are dividing, respectively (Edelmann et al., 2013). Also, in the amyloidosis model, aged Aβ42-treated zebrafish (18 months old) have increased radial glia proliferation, but still significantly less compared with young Aβ42-treated zebrafish (6 months old) (Bhattarai et al., 2017). This clearly highlights the effect of age-related stem cell exhaustion on the repair capacity of the aged teleost brain, which also results in a reduced production of newborn neurons (Bhattarai et al., 2017). In the young injured teleost telencephalon, newborn neurons express Tbr1, indicating differentiation into mature glutamatergic neurons, which was the original neuron type lost by injury (Kishimoto et al., 2012). Kroehne et al. (2011) confirm these results and spot newborn neurons at the injury site, in the parenchyma, and at the PVZ at 21 and 90 dpi, expressing NeuroD1, PV, mGlu2, and prox1, but also MAP2a + b and SV2, suggesting that these neurons are mature. Around 30–35 dpi, the young telencephalon is structurally regenerated, based on histology, neuron marker expression, and radial glial fiber distribution (Ayari et al., 2010; Kroehne et al., 2011; Kishimoto et al., 2012). No such data, however, exist for the aged injured telencephalon, yet they are indispensable in elucidating if newborn neurons can mature and integrate in the existing circuitry throughout the lifespan. In addition, to our knowledge, there are no reports describing recovery of function after injury in the aged teleost telencephalon. This represents a clear gap in knowledge (Figure 2, question mark). As it is clear that aging hampers neurorepair at the synaptic, inflammation, stem cell proliferation and neuron production level, does this then inherently also lead to incomplete repair and permanent functional deficits? Studying this is not straightforward and contradictory findings are often generated. In the zebrafish tail fin, heart (Itou et al., 2012), and optic nerve (Van houcke et al., 2017) and in the goldfish spinal cord (Bernstein, 1964), repair was delayed but in the end still functionally completed in the aged fish. Other reports on zebrafish, guppy, and killifish tail fin, however, show abnormal fin repair and a lower growth rate, and in the aged killifish, only 46% of the original tail fin size was observed at 27 days post amputation (Comfort and Doljanski, 1958; Anchelin et al., 2011; Wendler et al., 2015).

Overall, these reports agree that aging hampers or delays repair of tissue, and as such, it is fair to say that aged fish partly lose their regenerative ability. How this relates to the teleost brain remains, however, elusive. We believe that the long lifespan of zebrafish, medaka, goldfish, and other teleost fish (Figure 1) often discourages researchers to investigate this. In this regard, we anticipate a real benefit of using a new teleost model, the short-lived African turquoise killifish (*N. furzeri*), in future research. Its rapid aging and robust aging characteristics will expedite practical, fast, and clear investigations (Kim et al., 2016; Platzer and Englert, 2016).

## Conclusion

Inherently linked to our ever-increasing aging society is a rise in age-related neurodegenerative disease states. The irreversibility and the lack of therapy for such diseases impose a high socioeconomic burden on society. Its late onset and diverse nature more than ever point out the need for practical/efficient gerontology models. Preferably, a model with robust regenerative capacities should be used to address the limited neurogenic capacities of humans for the regrowth of neurons that are lost due to such diseases.

Studies employing aged teleost fish convincingly evidence that typical hallmarks of mammalian aging also exist in the aged brain of several teleost species. This strengthens their applicability as gerontology models, not only body-wide but also specifically for age-related neuropathology. As a consequence of brain aging, the teleost’s high regenerative ability is altered. It, however, remains unclear if these changes only cause a delay in the repair process or eventually result in permanent deficits. Hence, more research is needed to completely understand the impact of aging on teleost brain repair and how this can be exploited for the development of new therapies for successful recovery in the mammalian brain. We accredit teleost fish worthy of acting as a solid bridge between short-lived invertebrate and mammalian models for age-related pathology in vertebrates. In particular, killifish (*N. furzeri*) grasp our attention since their short lifespan and spontaneous neurodegeneration hold great promise to tremendously speed up and boost this research field.

## Author Contributions

JVH: conceptualization, writing, original draft, review, editing, and visualization. VM, CZ, ES, and AR: review and editing. LA: conceptualization, writing, review and editing. All authors listed have made a substantial, direct and intellectual contribution to the work, and approved it for publication.

## Conflict of Interest

The authors declare that the research was conducted in the absence of any commercial or financial relationships that could be construed as a potential conflict of interest.

## Abbreviations

CNS: central nervous system
DDR: DNA damage response
dpi: days post injury
DR: dietary restriction
hpi: hours post injury
mtDNA: mitochondrial DNA
NSC: neural stem cell
PVZ: periventricular zone
SA β -gal: senescence-associated β -galactosidase
SASP: senescence-associated secretory phenotype
TBI: traumatic brain injury
TERT: telomerase reverse transcriptase
VZ: ventricular zone.
